# Understanding therapeutic massage as a form of bodywork: knowing and working on the (energy) body

**DOI:** 10.1111/1467-9566.12814

**Published:** 2018-10-22

**Authors:** Jennifer Lea

**Affiliations:** ^1^ Geography University of Exeter Exeter UK

**Keywords:** Body, CAM (complementary and alternative medicine), Geography, Holistic medicine, Medical/healthcare workforce, Interviewing (qualitative)

## Abstract

Bodywork – as work which takes the body as its immediate site of labour – includes forms of service work, healthcare and caring. While work on bodywork has undeniably foregrounded the body, at the same time it has worked with a relatively limited understanding of bodily knowledges and practices. This article uses a theoretical framework taken from writing on Non‐Representational Theory, by Human Geographers, in order to take seriously ‘alternative’ body knowledge such as energy. The article draws on data from in‐depth interviews conducted with therapeutic massage practitioners in order to take seriously the ways in which energy directs and shapes the work that these bodyworkers do, adding new empirical understandings of what working with energy entails. It makes a broader conceptual contribution to bodywork literatures, advocating the importance of extending analysis beyond social constructionist approaches and questioning the taken‐for‐granted understandings of materiality that are most often drawn upon in order to attend to the kinds of knowledge that are less easy to formalise, anomalous, or that push at the fringes of the definite or the limits of the believable, but which are nonetheless central to many different kinds of bodywork contemporarily.

## Introduction

Given significance in the writings of sociologist Carol Wolkowitz, forms of bodywork take the body as the ‘immediate site of labour, involving intimate, messy contact with the (frequently supine or naked) body, its orifaces or products through touch or close proximity’ (2002: 497). In her agenda setting 2002 article, Wolkowitz argued that existing research had tended to neglect the *bodies* involved in bodywork. This was because it tended to either prioritise the idea of ‘emotional labour’ thus reproducing the mind–body dualism where the emotions were unproblematically located in the mind and the body was seen to merely produce ‘mechanical responses’ (2002: 499); or because it had focussed on the realm of discourse at the expense of the body. To address this neglect of the body, Wolkowitz outlined an agenda to focus attention towards the ‘*materiality* of body work practice’ (2002: 500, added emphasis), consisting of three suggestions for future research. The first of these was that researchers should attend to the ‘bodymaps’ and knowledges of the body that are articulated in texts and which are seen to ascribe a specific logic to the bodies involved in bodywork; second, focus should be directed towards the divisions of labour involved in bundling bodywork practices into higher and lower status forms and how this relates to power relations between working and worked upon bodies; and third, that the workplace interactions that shape the bodily relationship between bodyworker and client should be scrutinised.

Research on bodywork has responded to this agenda and has foregrounded the body; for example a 2011 special issue of this journal which aimed to develop Wolkowitz's work and to advance our understandings of bodywork. Here, Twigg *et al*. (2011) examined how the concept of bodywork might inform our current understandings of health and social care, Wainwright *et al*. (2011) looked at the regulation of bodies in the context of bodywork training, and Gale (2011) developed the idea of body‐talk to show how the body itself is seen to be able to communicate its needs and desires in the establishment of a therapeutic relationship. Gale's work foregrounds the actively communicating and knowledgeable body, thus problematising any notions of the passive patient that might circulate in writing on bodywork. The idea that we could usefully rethink the body as active and excessive in our accounts of bodywork forms the framework for this paper as it draws on the non‐representational agenda that has been set within human geography over the past 20 years or so. This literature explicitly re‐conceptualises the body as the subject (rather than object) of knowledge, and this in turn enables the expansion of the materialist imaginations that we deploy in our understandings of bodywork, so that they extend beyond the physicality of the flesh. This conceptual framework makes it possible for the paper to take more seriously how massage therapists, whose in‐depth interview transcripts provide the empirical material here, account for their work through ideas of energy.

Massage is categorised as a form of ‘complementary and alternative medicine’ (CAM), and can be understood as part of the development of contemporary cultures of wellbeing (Sointu 2006). The growth in CAM is seen by Stacey (2002: 270) to be one of the most ‘striking’ recent shifts in the field of health care in the UK. While energy is a key to the categorisation of massage as ‘complementary or alternative’, studies of bodywork have not tended to fully attend to the ways that energy is seen to shape massage as bodywork. The paper focuses on energy as a form of bodily knowledge, and the next section offers some definitions of energy and discusses how existing studies of bodywork have treated energetic knowledges. The section following outlines the recent work on non‐representational theory by human geographers that provides the framework for the paper, offering a discussion of first the relationship between body and knowledge, and second the materialist imaginations that might prove fruitful in future research into forms of bodywork. Following this, the case study and methods are introduced, and the paper then moves to present an account of the energetic knowledges used by the massage practitioners to narrate their work, and a discussion of the way that energy shapes the workplace practices of these bodyworkers, before concluding.

## Energy

**Figure 1:**
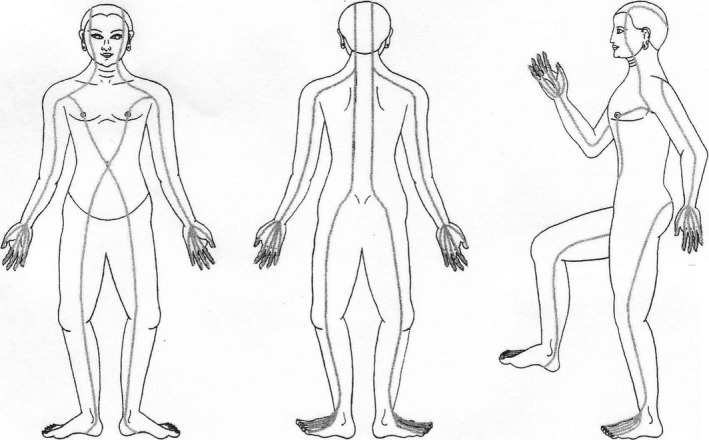
Diagrammatic representation of the Sen lines (from https://kuanthai.wordpress.com/category/sen-lines-2/ ‐ last accessed 8/8/18)

There are multiple understandings of energy that circulate in contemporary society. It is variously characterised through notions of bodily or mental tiredness and energy budgeting, a kind of atmospherics that is related to the feel of particular places, and the kind of bodily energy under discussion here (Philo *et al*. 2015). While different bodywork practices can differ considerably in their understanding of energy, and not all forms of massage have energy at their heart, it is useful to outline a basic sense of how energy is imagined and conceptualised in the kinds of massage that are discussed here. Also known by other name such as ‘ki’, ‘chi’ or ‘prana’, depending on the tradition drawn upon, the idea of energy is based on imaginations of an energetic flow around, through and beyond the body (see for instance Asokananda 1994). Maps have been produced of the energy lines that run through the body and extend out into the environment. These maps and the names of the lines differ depending on the traditions that the CAMs are drawing on (e.g. in Shiatsu they are called Meridians, in Thai Yoga massage they are called Sen lines – see Figure 1). When energy lines become blocked, or the flow becomes stagnated, illness and suffering are understood to occur. Practices such as massage are seen to work on the energy lines to remove blockages, to re‐establish free flow of energy, and to restore health.

At the same time as the mapped certainty of the energy body can be seen through representations such as these bodymaps, in practice there is an ambiguity about energy. Energy is easy to name, but also hard to describe and account for verbally or in written training manuals. There is lack of agreement about what energy feels like, with descriptions including the feeling of heat (without an external source), the fullness of a body, a pulsing or subtle vibration of the body or a sensation likened to the flow of water, for example (see Lea 2009: 472, Philo *et al*. 2015). In bodywork, energy can be described differently as it manifests in the practitioner's own body, or in the client's body. This inexact, inangible and unmeasurable quality of energy, and the ‘different attitude to the body and its boundaries’ (Twigg 2000: 397) that is enacted through the energetic, is one of the reasons why energy is positioned as an *alternative* to the dominant understandings of anatomy, physiology and pathology that form the basis of Western biomedical models of health and illness. Energy is one of the reasons why massage and associated practices are categorised as ‘complementary’ and/or ‘alternative’. The 2000 UK White paper on CAM notes clearly that the ‘abstract philosophy’ (of which energy is a key part) that lies at the heart of them, and for which is ‘no reasonable scientific basis’ is ascertainable, is problematic in the context of the kinds of ‘scientific reasoning and experiment’ (Science and Technology report 2000: section 2.17; House of Lords Select Committee 2000) which form the basis for dominant biomedical regimes of truth. Energy, and other alternative philosophies of life, have also proved a challenge for academic writing. For example, Doel and Segrott (2003: 743) argue that these ideas place much of the CAM milieu as somehow ‘beyond belief’, meaning that it is difficult to apprehend using the kinds of schemas and frameworks that are used in existing studies of the medical and health.

Energy has, however, received some attention in the context of bodywork, however. For example, work by Oerton (2004) and Oerton and Phoenix (2001) conceptualises energy as a discourse which allows intimate bodily contact in therapeutic massage to be defined as non‐sexual. In this analysis, the energetic realm is used by therapeutic massage practitioners to ensure that their work does not ‘impinge negatively on the individual's identity’ (Oerton and Phoenix 2001: 389). This is necessary, they argue, because of the wider social context where this kind of bodywork is assumed to involve sex. Here, energy becomes a narrative that creates a distancing from the corporeal body, such that in‐depth interview narratives were marked by ‘discursive landscapes that constitute the practitioner/client encounter as utterly disembodied’ (2001: 399). Energy takes on significance in Oerton's work *primarily* because of the symbolic work that it is seen to do in distancing therapeutic massage from the realm of sex. More recently, in her study of holistic massage practitioners, Purcell (2012: 212) notes that there is a conflict between the understanding of the leaky energy body that underpins massage and the dominant cultural constructions of personhood as ‘stable, bounded and autonomous’ (Lawton 1998: 134 in Purcell 2012: 212). While Purcell argues that seeing the body as ‘physically and energetically leaky’ is the main ‘sticking point’ for the ‘mainstreaming of Holistic Massage’ (2012: 212), she does not pay detailed or sustained attention to energy in relation to the *work* of bodywork. In a study of Homoeopathy, Gale (2011) argues that energy (alongside talk and observation) is part of the way that practitioners diagnose problems. Gale suggests that energy is part of a physical proximity between client and practitioner without necessarily making physical contact. She describes a relationship of co‐presence and attention between practitioner and client, where the Homoeopath shares the space of the consultation room and is a witness to each patient's emotions, through the cultivation of a ‘deeply embodied and reflexive approach that nurtures sensitivity to subtle changes in the atmosphere between two people’ (Gale 2011: 243). Gale points to the relational aspects of energy and the way it is set up between the bodies of patient and practitioner, but doesn't extend this discussion further. She describes energy as a form of touch without physical contact; a ‘non‐material’ form of touch (2011: 243).

Together, these show that some attention has been given to energy in studies of bodywork. To some degree, however, each of these treatments approaches energy through a lens of narrative or discourse, asking what it does to talk about the body through the idea of energy. Energy is taken to be a kind of symbolic device that performs a broader function to designate the work being as a particular kind, or to designate the identity, or the body, of the client and/or practitioner as a particular kind. At the same time, energy is designated as non‐corporeal and non‐material. This paper suggests that it is useful to develop an *additional* set of questions about the relationship between energy and bodywork by drawing on writing on the body and materiality. Rather than looking primarily at the social construction of the body, this work (outlined in the following two sections) suggests that we might also take a more *practice‐*based approach; drawing in a wider materialist imagination that does not simply assume that energy has a purely figurative role in the practice of therapeutic massage but instead interrogating the role it plays in shaping what practitioners *do*. In doing so, the paper makes a broader conceptual contribution to bodywork literatures, advocating the importance of extending analysis beyond social constructionist approaches and questioning the taken‐for‐granted understandings of materiality that we work with.

## Rethinking the body

Non‐representational theory was initially developed by human geographer Nigel Thrift (1996, 2007), and one of the things that this has prompted has been a renewed interest in, and problematisation of, the body among human geographers. With theoretical antecedents including phenomenology, neo‐vitalist thought and post‐structuralism (see Cadman 2009), non‐representational ideas foreground the relational body. Mobilising a geographical imagination that pays particular attention to the role of context, the body is seen as made and re‐made through its encounters. Rather than starting with a pre‐formed idea of what the body is, it is argued that we should instead ask how the body is ‘actualised as a part in an assemblage, or as a linkage of flows, as energies, agitations and intensities’ (Dewsbury 2000: 482). Furthermore, the ‘practical rather than cognitive’ (Thrift 1997: 126) are foregrounded, leading to a refiguration and expansion of what might be counted as knowledge such that the non‐cognitive and expressive are foregrounded. This paper draws specifically on a paper by Dewsbury (2000) that does work to specify what we might think of as a body. Here, Dewsbury argues that post‐Cartesian approaches to the body can be divided into those that take the body as the *object* of knowledge and those that take it as the *subject* of knowledge. Taking the body as the *object* of knowledge means that it is seen as a container (of our souls, personalities, brains, for example), or as a passive object to be inscribed with desires and wants (broadly the approach taken to energy described in the previous section). These approaches tend to take the body to be a self‐contained, whole organism that is ‘comprised and structured out of an intact, logically proportioned set of organs that bind its matter and energy flows neatly within unleaking ends’ (Dewsbury 2000: 482). The body as object is a tangible ‘thing’ that can be researched, conceptualised and explained. This description of the self‐evident fleshy body aligns with many of the accounts of the body that have underpinned studies of bodywork.

Dewsbury draws on the thinking of Deleuze and Guattari to outline the basis of an expanded imagination of the body (of the kind that characterises non‐representational thought). As the *subject* of knowledge, the body is seen less as a tangible ‘object’ and more as a connective space, constituted through its linkages to other bodies, objects and settings. This understanding approaches the body with different emphasis; rather than looking at the finished form of the body, it instead foregrounds the flows and connections that constitute the body from moment to moment. This means that we can add the ‘biological flow[s] of energy, matter, and stimulating chemical fluids’ (Dewsbury 2000: 485) into our picture of the body, asking how these relate to and complicate the fleshy corporeality of our existence. Bringing these bodily registers into academic accounts extends what we might think of as commonly accepted models of what counts as knowledge in social scientific accounts of the world (Dewsbury *et al*. 2002: 439–40). Approaching the body in bodywork as the *subject* of knowledge holds the potential to expand our understandings of the kinds of work might be done in the doing of bodywork, legitimising a consideration of the full range of ‘bodymaps’ and knowledges that are variously mobilised to account for different forms of bodywork. These kinds of knowledges – which might be less easy to formalise, are anomalous, or that push at the fringes of the definite or the limits of the believable – are nonetheless central to many kinds of bodywork. It also involves taking the body seriously as a creator of knowledges (not just something that can be known) across a range of bodily registers. Dewsbury argues that all ‘thinking, knowledge creating, and experience referencing is a bodily process’ (2000: 477), and this knowledge creating is central to the ways that we inhabit and are able to go on in the world.

Taking the body as *subject* of knowledge reframes the kinds of questions we might ask about the bodies involved in bodywork. For example, if we are less certain of the boundaries or form of a body, we might usefully ask what a body is, where it begins and where it ends (in this particular conjugation), stretching our understandings of the forms that bodies might take beyond anatomically ‘correct’ versions of the body. We might also be prompted to think beyond models of proximity which suggest that the most intimate form of contact is skin‐to‐skin contact, moving towards mixed bodies where intimacy extends into bodily interiors (Lea 2012). Our understandings of bodily wastes could be shifted beyond physical wastes (such as urine, faeces, vomit or blood) into other registers (e.g. bad energy), and as such we might begin to take seriously how the (negative) impacts of bodywork might be felt across multiple registers of the body. Relatedly, new understandings of practices of boundary‐making between practitioner and client bodies would need to be developed, which would move beyond attention to purely physical boundaries (e.g. latex gloves) to the establishment of other kinds of boundaries. As can be seen here, taking the body as *subject* of knowledge and letting things such as energy into our accounts has implications for our understandings of the materiality of bodywork practice (as framed through body–body relations, proximities and distances), and it is this that the paper turns to now.

## Rethinking materiality

Materiality is a key to the agenda set out for the future development of bodywork studies by Wolkowitz, but she says little about what is meant by materiality. The work on energy in studies of bodywork mentioned above brings questions of materiality to the fore, making a clear distinction between the (material) corporeal body and the (immaterial or non‐material) energy body. Here, there is a distinction implied between the real and unreal; the self‐evident and obvious solidity of the body, and the problematic and ambiguous invisibility of energy. While materiality remains unspecified in studies of bodywork, it can be taken to mean the flesh and bones of the body; the body as object of knowledge as coherent, obvious and unmistakably present. Again, non‐representational theory provides a resource to rethink this implicit approach to materiality. Anderson and Wylie (2009) problematise such implicit approaches to materiality on the grounds that they take matter for granted, leaving it unquestioned and self‐evident. They outline an alternative ‘material imagination’ which extends the material beyond simple physicality (2009: 319). The straightforward division between material and immaterial is undone, so that ‘materiality is never apprehensible in just one state’ but is rather ‘always already scored across states (solid, liquid, gaseous) and elements (air, fire, water, earth)’ (2009: 332). As well as including different states beyond the purely physical in conceptions of materiality, this approach sets the material realm in motion, suggesting that it is not ‘static or inert’ but rather ‘turbulent, interrogative, and excessive’ (2009: 332). Here, qualities that are usually associated with immateriality (e.g. liveness, movement, ephemerality) are seen to be ‘*of* matter, rather than standing in opposition to it’ (2009: 332, original emphasis).

In relation to the body and bodywork, then, this way of thinking materiality means asking how different bodily registers are implicated with each other; how might the body be emergent through the co‐implication of the flesh with the energetic, or the hormonal, or the affective? How does the body take place across different states, or become composed through the conjugation of different elements? What happens if, instead of casting energy aside as something somehow not real, or irrelevant, we reconfigure it as ‘internal to, rather than in supplement or opposition to, the taking place of matter and materiality’ (Anderson and Wylie 2009: 319)? If we choose not to see the energetic as somehow ‘obscuring’ the materiality of bodywork (Wolkowitz 2002: 500), then we might instead understand the energetic realm to be *of* the body. Having foregrounded the necessity and rationale for attending to energy, the paper moves on to introduce the study and methodology.

## The bodywork study

This article is based on qualitative research that aimed to develop an understanding of what was involved in heightened body practices such as massage, and how people were engaging with them contemporarily in the UK. The article draws on interview data with the aim of foregrounding the ways the practitioners narrated their own specific experiences of doing bodywork. Some of this interview data was collected as part of PhD research, which consisted of participant observation during a massage training course, a yoga retreat and in a healing space at a music festival, alongside semi‐structured in‐depth interviews with 10 Thai yoga massage practitioners. I conducted a second wave of 10 interviews with massage practitioners who worked across a wider range of types of massage (including Holistic massage, Swedish massage, Pregnancy massage, Aromatherapy massage, Indian head massage and Shiatsu) and other treatments (such as Zero balancing, Hopi ear candling, Reflexology and Reiki). These practitioners were recruited by writing to natural health centres in four major UK cities (Glasgow, Edinburgh, Bristol and London). Table 1 gives details of the interviewees whose narratives are drawn upon in this paper. This research was given approval by the research ethics committee at the University of Glasgow.

**Table 1:** Interviewees

Psuedonym	Location	Massage practice
Bob	Bristol	Thai yoga massage
Kerry	London	Thai yoga massage
Reuben	Bristol and Cheltenham	Thai yoga massage
Steven	Bristol	Thai yoga massage, Reiki, T'ui Na
Isabella	London	Thai yoga massage, zero balancing
Anna	Bristol	Thai yoga massage
Cara	London	Thai yoga massage, yoga teacher
Billy	Bristol and Totnes	Therapeutic massage
Grace	Glasgow	Thai yoga massage

One of the aims of the follow on interviews, in particular, was to explore what it was like for people who are skilled in working with the body to talk about the body; these were interviewees for whom the body was ever present in their work (both their own bodies and the bodies of clients), and I wanted to explore how far they had developed experience in talking about the body and articulating things about bodily experiences. On initial inspection, this decision to do further interviews (rather than more participant observation) contradicts the agenda that Wolkowitz set for the future of bodywork studies. She argued that researchers should employ participant observation to allow ‘sociological observations of the interactions of workers of various kinds with their patients, clients or customers’ (2002: 500) so that research might focus on the bodies involved in bodywork and give rise to data on the ‘largely implicit concepts of the body that circulate in actual workplaces’ (2002: 500) rather than being stuck in the realm of discourse though interpretations of texts relating to bodywork. While I do not want to suggest that Wolkowitz privileges observation of bodies at the expense of all other methods, it is useful to pay explicit attention to the role that interviews might play in drawing out ‘implicit’ concepts of the body that Wolkowitz suggests are important.

In relation to non‐representational theory, interviews have been afforded a somewhat ambivalent status, with researchers often arguing that interviews are not quite up to the job of capturing live action (Hitchings 2011). They have come to be problematised on the grounds that they happen ‘after the fact’ so can ‘only ever provide an unsatisfactorily washed out account of what previously took place (Thrift and Dewsbury 2000, in Hitchings 2011: 61). Other authors argue that interviews miss out on a whole raft of non‐representational and non‐representable aspects of life so we must be mindful of this limitation in our usage of them (MacPherson 2010: 8, in Hitchings 2011: 61). In response, Hitchings (2011) makes a clear case for the value of interviews in researching bodily practices, employing social practice theory to argue that people can indeed talk in quite revealing ways about their everyday bodily actions, even those which might be habitual or occur prior to conscious thought. I would re‐emphasise this in order to argue interviews have an essential role in expanding our understandings of bodywork, both on their own and alongside other research methods.

In this case, interviews allowed the practitioners to narrate their bodywork through the lens of energy. This brought a key aspect of their practice to attention, which would not have happened if participant observation had been the only method. For example, if I had relied only on the participant observation I did in the healing space of the music festival I would not have recorded anything about energy in my research diary as it wasn't visible. Again, if the research had relied solely on observation of practitioners (which is hard to do in the usual closed spaces where massage therapy takes place), energy might not have shown up because of this lack of visibility, and also because the practitioners often do not talk to clients about energy. While energy did feature in the research diary documentation of my own learning of Thai massage, my experience as a beginner was tied very closely to where the lines were mapped out on the body (in some ways just reproducing the textbook knowledges that I was learning) and I could not say that I felt the energy lines in any tangible or certain way. In contrast, the energetic realm was narrated clearly and with certainty during the interviews conducted with experienced massage practitioners. I explicitly asked about energy and its relationship to other kinds of knowledge, for example the anatomy and physiology qualifications which must be undertaken as part of training in order to gain insurance. I also explained that I had been learning Thai massage as part of my research. This gave us a shared vocabulary and perhaps allowed them to make some assumptions about the legitimacy and value I placed on ideas of energy. As such, interviews can have an important role in the understanding of bodywork as they can open up discussion of registers that are not necessarily obvious in practice, but which are nonetheless of central importance to the doing of bodywork. The paper now turns to the interviews themselves to develop an understanding of the role of energy in defining the body.

## Defining the body in therapeutic massage

The definitions, or ‘bodymaps’, that ascribe a specific logic to the body (or bodies) involved in bodyworking are important to the agenda that Wolkowitz sets out. As the previous section suggested, Wolkowitz advocates observational work to flesh out the ‘more implicit concepts of the body’ (2002: 500) that are important in bodywork, and this paper demonstrates the value of interviews in this task too. Taking account of the bodyworker's accounts of the body and knowledge opens up opportunities to take the body as *subject* of knowledge; as a creator of knowledges across a range of bodily registers. The interview narratives in this section tap into the practitioner relationship with the body and its knowledges; in particular how they use energy as a way of creating knowledge, how energy is a form of knowing, and how they use this bodily knowledge to shape their engagement with the client. While energy is a contested knowledge in the wider field of health, the interview narratives under discussion here showed that energetic definitions of the body structured the training and learning of the massage practitioners.^1^

The interviews reflect the fact that the bodyworkers saw energy as a legitimate way to narrate and explain their work, being something that they felt as ‘real’, which they had begun to develop a language to talk about, and which formed the basis of the relation set up between their bodies and their client's bodies. This was true to such a degree for some practitioners that they explained their work to me solely in terms of energy. For instance, Bob explained that, while he had done the required anatomy and physiology (A&P) course as a separate qualification, his practical massage training ‘didn't include anatomy or physiology … and erm it's all purely based on energy lines, Sen lines and the sense of touch and movement – there was just no Western scientific input’. Similarly, Kerry told me that the massage training that she had done was structured totally around energy rather than anatomy and physiology: ‘Thai massage strictly speaking doesn't follow A&P and it isn't taught alongside [energy] because you're not working on the muscles, you are working on an energy system’. Reuben told me that the theoretical part of his course dealt solely with the idea of energy, noting that every morning ‘there was theory so you were looking at … the energetic lines’. These three interviewees had all trained in the same lineage of Thai yoga massage where energy was the key knowledge taught about the body (see Lea 2009: 468), and for them, energy had become the most significant body knowledge through which they approached and understood their bodywork.

For other interviewees, knowing the body via energy was not enough and they told me that they also drew on other knowledges to assemble a ‘working knowledge’ of the body. The most common of these was an energetic understanding of the body, combined with biomedically based A&P knowledges. Steven described how he put together his energy and A&P knowledges of the body to understand his practice. He described the energy body to me in concrete and definite terms:

it's an energy that you can feel, and you've got to remember that whether it's a Sen line or a meridian it, you know, lies deep in the body. They're not superficial or surface lines so they lie underneath the muscle, soft tissue and in some cases, you know, ribs, organs. And, you know, some cases can't literally be palpated so it's an extension that's needed to sense them underneath.

However, he said he could not use massage therapeutically (using the massage to ‘actually heal problems and, you know, look at treating specific injuries’) without his A&P knowledges. In addition to his Thai massage training, Steven had undertaken more advanced study in anatomy and physiology, and training in T'ui Na – a form of Chinese massage^2^:

the only way for me to be able to use Thai massage as a healing tool and actually gaining an understanding of the body is to you know, you know have quite a deep understanding of Western anatomy on top of that as well. And also look into, further into Chinese medicine from where it takes some of its roots.

Isabella was less emphatic about the importance of A&P knowledges. She described how she, and many of the people who had been on the same training courses as her, had developed a really tangible sense of what energy feels like and how it moves around the body:

there are some people I've trained … alongside, who have definitely developed a good sense of where those energy lines are – they can feel the energy. And I've been through stages of where I can feel the energy very clearly in these lines.

She went on to describe how, by approaching the body through energy, she developed a sensitivity to the body she is working on:

I did a body massage course and I did anatomy and physiology because I knew I needed … knowledge of the anatomy and physiology. ‘Cause Thai massage strictly speaking doesn't follow A&P and isn't taught alongside because you're not working on the muscles, you are working on an energy system. I mean we kind of did the skeleton – you know, these are the things you've got to look out for: if it's sprained or strained. So in terms of injuries and which muscle inserted where, [I have a] very, very general idea and I've always felt that I worked much more like: ‘ok, I actually do not know what that muscle is called and whether it inserted there or not’, but it's a bit like ‘well how does it feel?’ So I've always worked in a much more hands on: ‘let's take it really slowly, let's breathe, tune into how it feels and lets find a way of releasing it’, rather than having a picture in my mind of ‘those are the muscles – I know I have to release those first’.

What emerges here is an account that suggests that energy operates as a way of directing the attention of the practitioner towards the body of the client, enabling a finding out of how that body is. During training, the practitioners are encouraged to feel the energy through their touch, and to ‘develop a “feeling” for the energy flow through the body’ (Lea 2009: 471). While Gale (2011) notes that energy might be a way of understanding proximity without touch, here touch is integral to the kinds of diagnostic and healing touch that Isabella uses.

Energy is, therefore, not just a way of *knowing the body*, but also offers a way of *knowing through the body*. It can be seen as a form of intelligence that comes from the body (in relation) and which affords a felt sensitivity and attention to what the body of the client presents. Energy offers a language for the practitioners to articulate the work of tuning in, or ‘becoming sensitive’, to what the body of the client presents. This language shifts understandings of the body of the client from passive to active; through the energetic register, the problematic body can transmit information to the practitioner who is able to work energetically (similar in some ways to the forms of body‐talk outlined by Gale (2011)). Energy also provides a register through which the practitioner might make an impact on the body of the client and offer treatment. In these interview narratives, energy is understood to be *of* the body, rather than in opposition to it. Rather than obscuring the body, energy is part of the body; important in understanding and treating the problems it might hold. To sum up this section, the interview narratives suggest that, to different degrees and in different ways, energy is a significant knowledge in the practice of massage as bodywork. It is significant in narrating the bodies involved, understanding the work done, and the effects of the massage. While some practitioners saw it as an alternative to anatomical and physiological bodymaps, most of the practitioners described their understanding of the body as a kind of hybrid knowledge formed from energetic and more conventional anatomical knowledges. As well as energy forming a bodymap through which the body becomes known, a sense that energy offered something to know‐with emerged. Energy was articulated as a form of bodily intelligence; a way of attending to and decoding the problems of the client. These interview narratives underline the value of moving beyond analysis of the body as known within training manuals or books, towards understanding the body as knowledgeable in itself.

## Workplace interactions: proximities and distancing

In contrast to other forms of service employment, co‐presence is essential to forms of bodywork when the ‘object of work is the physical manipulation of the body of a customer, client, or patient’ (Cohen 2011: 195). In looking at workplace interactions, Wolkowitz emphasises the relations of proximity and distance between bodies. In many cases, bodily proximity is essential to the work being done, for instance Twigg's (2000: 402) study of caring for (ageing) bodies in which ‘direct physical contact, access to nakedness and the sharing of bodily processes’, are necessitated. Such close relations are ‘powerful mediators of intimacy, containing a capacity to create closeness and dissolve boundaries between people’ (2000: 402–3). Often, however, this intimacy is problematic as the body of the client is seen to be polluting, dirty and transgressive, and bodyworkers who are unable to employ distancing techniques are left to deal with the messiness of flowing, seeping and leaking bodies and to perform much of the physical labour involved in moving, touching and being physically proximate to the bodies worked on. A range of distancing techniques have been found to exist within bodywork, including the use of humour, using gloves (which not only provide a physical barrier, but also do work in putting up a symbolic ‘barrier of professionalism between the client and the worker’ (Twigg 2000: 404)), and hiding this kind of ‘dirty work’ in spatially segregated (private) spaces.

This kind of bodily proximity is integral to the therapeutic work of massage. While much of the discussion of proximity and massage has focussed on the sometimes ambiguous relationship between massage and the sexualisation of labour (see Oerton 2004, Oerton and Phoenix 2001, Purcell 2013) there has been some consideration of the effects of working in close proximity with the cared for body. Wolkowitz (2002: 505) notes that massage practitioners are likely to be working with ‘clean, cared‐for, continent bodies’ so will feel less need to ‘distance themselves from the polluting body fluids … that routine nursing work involves’ (Wolkowitz 2002: 505). Pollution here can be seen to be a quite literal thing in relation to human wastes and dirt and the management of feelings of disgust related to these (Twigg 2000: 395). Attending to energy through the interview narratives refigures these understandings of proximity, distancing and pollution. The practitioners interviewed did see the clients’ bodies as potentially polluting and understood this pollution in energetic terms. For example, Anna, Reuben, and Cara told me that they had experienced being quite strongly affected by the transfers ongoing between bodies, describing them variously in terms of energetic and forceful transfers:

because you tap into someone else's energy, like you would in everyday life, there's people who really drain you when you're with them, and they just seem to vampire all your energy and your positivity (Anna).

often I will leap up these stairs here after a massage, and I'll come out feeling like a leopard or something. I'll be energized. It's very rare that I'll come away from a massage feeling deflated, it can happen if I'm, if I do not protect myself when I'm giving a massage, and the person is *full* of negative energy (Reuben, his emphasis).

if you're putting force into somebody's body … they're receiving it … how much that person's learnt to receive determines how much you're going to get back. So, if they're resisting you and you're not quite trained enough or experienced enough to know how much force you should be putting in to meet that resistance, you're going to be getting that resistance back (Cara).

These descriptions characterise the clients’ and practitioners’ bodies as open to each other and receptive to each other's energy which is transmitted via touch. The practitioners accounted for the effects of their work via energy and the negative effects of close touch were not narrated in terms of skin‐on‐skin contact (e.g. dirt, smells), but rather in terms of the contaminating effect of the clients’ energy.

This alternative model of proximity (in which bodies are radically open to each other, and their energies mingle) problematises the model of proximity and distance that is based on *actual distance* between bodies which has thus far been used by researchers writing about bodywork. While bodies might be closely touching, the kinds of proximity that the massage practitioners are concerned with here go beyond the skin and into the bodily interior. The practitioners understand their bodies to become radically mixed and mingled with the bodies of their clients, via the flow of energy between bodies. Here, the proximities involved in touch might go beyond touch on the outside of the body, and extend into the interior via the flow of energy (see also Lea 2012). This had tangible effects for the practitioners, for whom the lingering, and potentially negative, effects of the clients’ energy were seen as a threat. It also had clear consequences for the doing of the bodywork, in particular the kinds of distancing techniques they saw to be appropriate. A range of distancing techniques were employed, which each attempted to maintain the integrity of the practitioner and their energetic separation from the client. For example, Billy told me about the visualisation techniques that he used before he started massaging:

what I try to do at the beginning of each treatment is to protect myself so imagine a white light around me or imagine some sort of barrier between me and the person so you do not actually take on their energy. Because a lot of, most of my treatments are energetic, although they're physical, they are energetic. So you take on people's energies and I've learned through, to my own detriment that you take, you can take on people's stuff … So I've got to protect myself so … unless I'm sort of rushed I tend to protect myself before each treatment so that I do not take on board people's problems and energies.

Here, he describes the use of visualisation to try to create an energetic separation between himself and his client. Similarly, Anna told me that visualisation was useful, but in a slightly different way: ‘I think it's just protection against it … you also have to detach yourself and look at it as … giving and trying as much as I can and then you have to switch yourself off and go to the next thing you have to do’. Here, Anna describes the use of visualisation techniques to enable her to ‘detach’ and ‘switch off’ from the energetic effects of doing massage as bodywork. While at first glance, Billy and Anna could be seen to be talking about energy symbolically, what they were actually describing were the ways that energy is, for them, a tangible ‘thing’ that they can feel and that is registered through their bodies (both in the immediacy of their touch, and as a lingering effect), and that forms the basis of their distancing and separating strategies. They talk about the possibility of energetic transfers from their clients as having a lasting effect, enduring through time and space, to have an effect on their lives *outside* their work.

Other practitioners echoed these concerns, but described how they used their work places to help create a distance between their working and home lives to stop the potential energetic pollution of their lives outside of work. For example, Grace told me:

I do not massage at home unless the person is a friend or it's somebody … I feel really needs it because I do not have a separate clinic room at home … So I've got a business space … And now it's like people do tell me stuff and I hear it and *I hear it in the room and I won't really think about it until the next time I see them*. ‘Cos that's where I put it, because otherwise what I found was, when I was doing stuff at home when I was training I would sometimes not sleep. I'd be thinking I wonder what's happening there, or what's going on … I mean great for case studies *but you do not want that energy in your house* (added emphasis).

Grace also described the cleansing of any negative energetic traces that might linger in the massaging workplaces. She told me:

I burn [essential] oils and … it tends to be, not always, depends what mood I'm in but *often it's basil because it's head clearing and space clearing* and I'll do it with something like grapefruit which is energising or juniper … so that's a smell that tends to be in my room (added emphasis).

Doel and Segrott (2004: 731–2) note that because of their ‘agency and expressiveness’, essential oils are prized within the therapeutic encounter of a range of different forms of CAM. Doel and Segrott understand them through their potential action to ‘transform bodies, and enable particular kinds of embodied experience to take place’ and their ability to ‘produce particular forms of embodied experience and affect’ (2004: 730), as well as through their ability to transform space (2004: 747). Wainwright *et al*. (2011: 222) have also noted the ‘sensuous transformation’ that can be effected by changing lighting, sound and ambience in order to create a ‘restorative environment for the client’. What Grace's quote suggests is that they do not just have an effect for the client but also for the practitioner who uses the olfactory and symbolic aspects of specific essential oils to manipulate the clinical space such that energy traces are cleared.

In addition to these distancing techniques, the practitioners also described cleansing techniques they used before and after doing bodywork. The notion of cleansing the working body has had limited purchase in the bodywork literature (perhaps because of the focus on cleaning client bodies, and the use of physical barriers such as gloves where there is a risk of body fluids leaking out), although Oerton (2004) underlines the symbolic value of the clean practitioner body, which plays a part in constituting a reputable professional identity and in distinguishing therapeutic massage from sex work. The practitioners here described their cleaning practices during and following a massage. These were based both on a conventional model of hygiene (they described their use of wipes and sanitiser on their hands and the client's feet in order to cleanse them before touch), and also in energetic terms (e.g. using techniques such as flicking their hands and wrists throughout the massage to let go of the energy of the client and using their hands to sweep the energy traces from their bodies after the massage). Energy, then, both on its own and as part of a hybrid form of knowledge about the body, plays a large part in the doing of bodywork, impacting on the ‘material experiences of interacting with the bodies that form the site of work’ (Wolkowitz 2002: 504). These discussions of workplace practices of distancing and cleansing show that the practitioners don't see energy as somehow separate from these ‘material experiences’, as ‘beyond belief’, or as separate from the corporeal body. Instead, the energy flows are implicated in the materiality of doing bodywork, providing the basis for organising the space of the workplace and the ways that the practitioners look after themselves, mediating the negative effects of the work they do. Rather than excluding energy (and other similar body knowledges that have been seen as immaterial or lacking in reality) from studies of bodywork on the basis of calls to attend to materiality, employing an extended understanding of what we might mean by materiality has provided valuable in understanding the working practices and workplace interactions of these massage practitioners.

## Conclusion

Attending closely to different forms of bodywork raises important questions about what the body is, how it is configured and known by workers, how it can be manipulated and changed, and towards what ends this change is directed. Studies of bodywork are of continued significance given the wide range of occupations that can be seen to be forms of bodywork, the cultural, social and economic significance of the category, as well as the central position that bodywork holds in the formation of contemporary embodied identities. Wolkowitz's work continues to provide a keystone for researchers who are interested in the practice and function of different forms of bodywork. This article drew on interview data from massage practitioners to begin to develop a better understanding of the kinds of body knowledges they use to underpin their work (namely energy), and how these knowledges relate to their material practices. These accounts of energy necessitated the adoption of a conceptual framework which opened up space to attend to the excessive, ambiguous, and challenging nature of the energetic realm. At the same time, however, the empirical materials presented here contribute to conceptual agendas within studies of bodywork by conveying how bodies are always much more than just passive objects. The paper argues that bodies should be understood as active and knowledgeable, as the interviewees invoke particular registers (which challenge many of our received understandings of the world) in their accounts of bodywork. Working with that which is generally taken to be ‘beyond belief’ or somehow metaphorical is necessitated if we are to value these accounts generously offered by the massage practitioners, even if they don't directly fit with our own understandings of the world. While we might intuitively situate knowledges such as energy as outside the realms of the possible, the presence of energy in the interview narratives presented here demanded that a framework be developed in which they could be taken seriously.

The paper makes a case for the use of interviews in studies of bodywork, as they open up wider accounts than simply doing textual analysis of books and training manuals, or doing participant observation. The interviews here proved particularly valuable because they set out a clear role for energy in the doing of bodywork. Had energy not entered into the interviews themselves, or been set aside in the analysis, the account of the knowledges used by the practitioners in their bodywork practice would have been incomplete. The interviews made clear the role of energy in practice; rather than being solely a framework through which the body became known, energy was understood to be a knowledge‐with‐which the massage practitioners did their bodywork. This in turn allowed the development of understandings of the body of the practitioner as a knowledgeable and sensitive relational space. It is not enough for accounts of knowledge in bodywork to dwell solely on the bodymaps that are seen to structure the worker's understanding of the client. Instead future accounts of bodywork need to offer accounts of the knowledges that the working body enacts, taking all the bodies involved in bodywork to be knowledgeable (moving beyond the higher status forms of bodywork and the biomedical knowledges that afford them that status, and including lower status forms of bodywork and the bodies of clients), and challenging our received understandings of what counts as knowledge.

The paper took Wolkowitz's demand that we attend to the materialities of bodywork seriously. The definition of materiality has remained implicit in studies of bodywork so far. Prompted by writing on energy in studies of bodywork, the paper set to problematise the dualism set up between the immaterial or non‐material energetic realm, and the material fleshy body. This opens up future research agendas in work on bodywork, which might interrogate the relationship between different bodily registers, the ways that forces or flows that might previously have been cast out as immaterial actually shape the work and workplace relations of bodywork, and how these expanded understandings of materiality might refigure the very basis of understandings of proximity and distance that underpin the current divisions of bodywork into higher and lower status. These expanded imaginations of the body and the kinds of materialities that might be involved in bodywork, offer a powerful lens through which to rethink what bodyworkers do, how they organise their work and workplaces, and what the *work* in bodywork entails. They also have broader consequences for studies of health and illness, as they direct our attention towards a fuller range of vocabularies and ways of knowing that are becoming more prevalent in contemporary understandings of the self, health and wellbeing, both in specific health‐giving encounters and in everyday life more generally (Philo *et al*. 2015). The paper sets a clear agenda for social scientists to begin to account for and interrogate these important ‘alternative’ bodily knowledges.

## Data Access Statement:

The data was collected prior to changes in the ESRC's research data policy. The dataset is not available because it was destroyed following analysis as agreed in the consent process.

## Notes

1 Taking account of the interviews alongside the participant observation problematises this statement somewhat, because a difference exists between how the practitioners understand and narrate the work that they do in the interviews (energy figures prominently), and how they account for their work to their clients (energy often doesn't figure). This invites questions (beyond the scope of this paper) about the kinds of knowledges that are legitimate in different contexts, and why, and perhaps also speaks to the conflict between the leaky energy body and the culturally dominant constructions of personhood as stable and autonomous that Purcell (2012) outlines.

2 This kind of training in multiple forms of complementary and alternative medicine was common amongst the interviewees, partly because of the system of continued professional development required by the insurance companies which demands that CAM practitioners undertake training every year.

## References

[shil12814-bib-0001] Anderson, B. and Wylie, J. (2009) On geography and materiality, Environment and Planning A, 41, 318–35.

[shil12814-bib-0002] Asokananda . (1994) The Art of Traditional Thai Massage. Bangkok: Editions Duang Kamol.

[shil12814-bib-0003] Cadman, L. (2009) Nonrepresentational theory/nonrepresentational geographies In Kitchen R. and Thrift N. (eds) International Encyclopaedia of Human Geography. Oxford: Elsevier.

[shil12814-bib-0004] Cohen, R. (2011) Time, space and touch at work: body work and labour process (re)organisation, Sociology of Health and Illness, 33, 2, 189–205. 21299568 10.1111/j.1467-9566.2010.01306.x

[shil12814-bib-0005] Dewsbury, J.D. (2000) Performativity and the event: enacting a philosophy of difference, Environment and Planning D, 18, 473–96.

[shil12814-bib-0006] Dewsbury, J.D. , Harrison, P. , Rose, M. and Wylie, J. (2002) Enacting geographies, Geoforum, 33, 4, 437–40.

[shil12814-bib-0007] Doel, M. and Segrott, J. (2003) Beyond belief? Consumer culture, complementary medicine, and the disease of everyday life, Environment and Planning D, 21, 739–59.

[shil12814-bib-0008] Doel, M. and Segrott, J. (2004) Materializing complementary and alternative medicine: aromatherapy, chiropractic, and Chinese herbal medicine in the UK, Geoforum, 35, 727–38.

[shil12814-bib-0011] Gale, N. (2011) From body‐talk to body‐stories: body work in complementary and alternative medicine, Sociology of Health and Illness, 33, 2, 237–51. 21029118 10.1111/j.1467-9566.2010.01291.x

[shil12814-bib-0012] Hitchings, R. (2011) People can talk about their practices, Area, 44, 1, 61–7.

[shil12814-bib-0013] House of Lords Select Committee . (2000) Science and technology sixth report. Available at http://www.parliament.the-staionery-office.co.uk/pa/ld199900/ldselect/ldsctech/123/12301.htm (Last accessed 08 August 2018).

[shil12814-bib-0014] Lea, J. (2009) Becoming skilled: the cultural and corporeal geographies of teaching and learning Thai yoga massage, Geoforum, 40, 3, 465–74.

[shil12814-bib-0015] Lea, J. (2012) Encountering touch: the ‘mixed bodies’ of Michel Serres In Paterson M. and Dodge M. (eds) Touching Space. Andover: Placing Touch, Ashgate.

[shil12814-bib-0017] Oerton, S. (2004) Bodywork boundaries: power, politics and professionalism in therapeutic massage, Gender, Work and Organization, 11, 5, 544–65.

[shil12814-bib-0018] Oerton, S. and Phoenix, J. (2001) Sex/bodywork: discourses and practices, Sexualities, 4, 4, 387–412.

[shil12814-bib-0019] Philo, C. , Cadman, L. and Lea, J. (2015) New energy geographies: a case study of yoga, meditation and healthfulness, Medical Humanities, 36, 1, 35–46. 10.1007/s10912-014-9315-3 PMC4352603 25503269

[shil12814-bib-0020] Purcell, C. (2012) Touching work: a narratively‐informed sociological phenomenology of holistic massage. Unpublished PhD thesis, University of Edinburgh.

[shil12814-bib-0021] Purcell, C. (2013) Touch in holistic massage: ambiguities and boundaries In Wolkowitz C., Cohen R., Sanders T. and Hardy K. (eds) Body/Sex/Work: Intimate, Embodied and Sexualised Labour. Basingstoke: Palgrave Macmillan.

[shil12814-bib-0023] Sointu, E. (2006) The search for wellbeing in alternative and complementary health practices, Sociology of Health & Illness, 28, 330–49. 16573719 10.1111/j.1467-9566.2006.00495.x

[shil12814-bib-0024] Stacey, M. (2002) Concluding comments In Bedelow G., Carpenter M., Vautier C. and Williams S. (eds) Gender, Health and Healing: The Public/Private Divide. London: Routledge.

[shil12814-bib-0025] Thrift, N. (1996) Spatial Formations. London: Sage.

[shil12814-bib-0501] Thrift, N. (1997) The Still Point: resistance, expressive embodiment and dance In Keith M. and Pile S. (eds) Geographies of Resistance, pp. 124–51. London: Routledge.

[shil12814-bib-0026] Thrift, N. (2007) Non‐Representational Theory: Space, Politics and Affect. London: Routledge.

[shil12814-bib-0027] Twigg, J. (2000) Carework as a form of bodywork, Ageing and Society, 20, 389–411.

[shil12814-bib-0028] Twigg, J. , Wolkowitz, C. , Cohen, R. and Nettleton, S. (2011) Conceptualising body work in health and social care, Sociology of Health and Illness, 33, 2, 171–88. 21226736 10.1111/j.1467-9566.2010.01323.x

[shil12814-bib-0029] Wainwright, E. , Marandet, E. and Rizvi, S. (2011) The means of correct training: embodied regulation in training for body work among mothers, Sociology of Health and Illness, 33, 2, 220–36. 21054441 10.1111/j.1467-9566.2010.01287.x

[shil12814-bib-0030] Wolkowitz, C. (2002) The social relations of bodywork, Work, Employment and Society, 16, 3, 497–510.

